# Cognitive Functions in Patients with Moderate-to-Severe Obstructive Sleep Apnea Syndrome with Emphasis on Executive Functions and Decision-Making

**DOI:** 10.3390/brainsci13101436

**Published:** 2023-10-10

**Authors:** Lei Zhao, Yanyan Zhao, Dongmei Su, Zhi Lv, Fei Xie, Panpan Hu, Kierstin L. A. Porter, Isabella Mazzei, Jaeson D. Chin, Yongsheng Wang, Yujiang Fang

**Affiliations:** 1 Department of Respiratory Medicine, The Second People’s Hospital of Hefei, Hefei Hospital Affiliated to Anhui Medical University, Heifei 230011, China; 19955873766@163.com (L.Z.); m18949678116@163.com (Y.Z.); sudongmeihuxi@126.com (D.S.); lvzhi0217@126.com (Z.L.); 2 Neurology Department of Neurology, The Second People’s Hospital of Hefei, Hefei Hospital Affiliated to Anhui Medical University, Heifei 230011, China; zft_xs@163.com; 3 Department of Neurology, The First Affiliated Hospital of Anhui Medical University, Heifei 230022, China; hpppanda9@126.com; 4 Department of Microbiology, Immunology & Pathology, Des Moines University, Des Moines, IA 50312, USA; kierstin.l.porter@dmu.edu (K.L.A.P.); isabella.mazzei@dmu.edu (I.M.); jaeson.d.chin@dmu.edu (J.D.C.); 5 Department of Surgery, University of Missouri School of Medicine, Columbia, MO 65212, USA

**Keywords:** obstructive sleep apnea syndrome, cognitive function, Game of Dice Task, verbal fluency test

## Abstract

Background: Patients with obstructive sleep apnea syndrome (OSAS) have cognitive dysfunction in many aspects, however, these patients’ decision-making function remains unclear. In this study, the Game of Dice Task (GDT) was used to investigate the function of decision making in patients with OSAS. Methods: 30 participants with moderate to severe OSAS and 27 participants with no or mild OSAS diagnosed by sleep breathing monitor were selected from June 2021 to March 2022. Risky decision making was tested through the GDT with known risk probability. General demographic information and background cognitive functions, such as the overall cognitive functioning and executive functioning, were tested to establish baseline data. Results: There were no significant differences in gender, age, and years of education between the two groups. During the GDT, the moderate to severe OSAS group opted for the safety option at a statistically significant lower rate when compared to the no or mild OSAS group (7.53 ± 4.43 vs. 10.26 ± 4.26, *p* = 0.022). The moderate to severe OSAS group utilized the higher risk option than the group with no or mild OSAS (10.47 ± 4.43 vs. 7.74 ± 4.26, *p* = 0.022). The utilization rate of negative feedback in the moderate and severe OSAS group was lower than that in the no or mild OSAS group (7.50, 52.50 vs. 28.57, 100.00, *p* = 0.001). At the end of the GDT, the moderate and severe OSAS group was more likely to have negative total assets than the patients with no or mild OSAS (−1846.67 ± 2587.20 vs. 300.00 ± 1509.97, *p* < 0.001). Multiple linear regression analysis shows that there is a negative correlation between the selection of risk options and negative feedback utilization in the GDT. Conclusion: Patients with moderate and severe OSAS displayed impaired decision-making throughout the study. Impaired decision-making is related to executive processes and may be caused by diminished prefrontal cortex functioning. However, the functions of memory, attention, language, abstraction, and orientation are relatively retained.

## 1. Introduction

Obstructive sleep apnea syndrome (OSAS) refers to the occurrence of airway obstruction and dyspnea during sleep, resulting in chronic intermittent hypoxemia, elevated blood carbonic acid levels, and abnormal sleep structure [1]. This sleep respiratory disease eventually leads to a series of pathophysiological changes in the body. About one billion people between the ages of 30 and 65 are affected by OSAS, of which about 425 million are thought to have moderate to severe OSAS [2]. Studies have shown that more than 40% of patients with OSAS have mild cognitive impairment, such as forgetfulness, difficulty concentrating, and abnormal decision-making ability, all of which affect the quality of life for patients [3,4]. The more severe the degree of OSAS, the greater the possibility of cognitive impairment [5].

Decision-making is a function of cognitive ability during which people solve problems in distinct ways through an interplay of personal experiences, realistic environmental conditions and their perception of the developing scenario [6]. The ventral medial prefrontal cortex, orbitofrontal cortex, ventral striatum, and amygdala have all been implicated in the function of decision making [7]. The Game of Dice Task (GDT) is often used to measure the function of decision making regarding known risk probability; however, this risk probability has not been well studied in patients with OSAS.

## 2. Materials and Methods

### 2.1. Participants

Patients who were admitted to the Department of Respiratory and Critical Medicine and Otorhinolaryngology head and neck surgery of Hefei Second People’s Hospital from June 2021 to March 2022 were selected for sleep breathing monitoring due to snoring. The participant ages ranged from 18 to 65 years old. All participants completed a minimum of primary school. They were then matched in sex, age and years of education. According to the results of respiratory sleep monitoring, 30 patients were allocated to the moderate and severe OSAS group, and 27 patients were allocated to the no or mild OSAS group. The diagnostic criteria of OSAS are as follows: oral and nasal airflow ceases for more than 10 s throughout the entire monitoring process, 30 or more apneic events [reduction of airflow greater than or equal to 90% compared to the pre-event amplitude [8] or apnea-hypopnea index (AHI) ≥five times/hour throughout testing. An AHI ≥ 15 times/hour is required for moderate and severe OSAS [9]. All patients completed cognitive tests on the night of sleep monitoring. This study was approved by the Ethics Committee of the Second People’s Hospital of Hefei (2022-ky-094), and all the observers provided informed consent.

Subjects were excluded from this study if they had previous cognitive impairment or diseases that alter cognitive function, such as a new cerebrovascular accident, massive cerebral infarction or cerebral hemorrhage, craniocerebral trauma, mental illness, or hypothyroidism. Subjects could not participate in this study if they have a diagnosis of mixed or central sleep apnea. Patients with acute or critical illness, including pregnant or lactating women, or severe heart, liver, or kidney diseases were also excluded from this study. Subjects were excluded if their MMSE total score was assessed as positive dementia, or a total score of less than 17, those who are in elementary schools who do not score more than 20, and those who are in junior high school and above who score no more than 24. Finally, those with failure or inability to cooperate with this study were excluded as well.

### 2.2. Data Collection

All patients underwent sleep breathing monitoring, and a sleep monitoring report was issued by standardized qualified health care professionals to analyze the results. Each questionnaire was completed by all participants.

### 2.3. Questionnaires

All participants completed cognitive questionnaires and Game of Dice Task experiments on computers under the guidance of trained clinicians.

The basic cognitive function of each participant was assessed using the Montreal Cognitive Assessment (MoCA) given its high rate of specificity and sensitivity [10]. The MoCA requires a total of 20 min to complete, divided into 10 min of testing followed by 10 min of rest. The MoCA encompasses several subscores, including visuospatial/executive function (5 points), naming (3 points), delayed recall (5 points), attention (6 points), language (3 points) abstraction (2 points) and orientation to date, month, year, day, place and city (6 points). A total possible score is 30. A total score of 1 was added to subjects with an education level of 12 years or less. Patients who scored 26 or more are considered to have normal cognitive functioning. Anything below 26 is considered cognitive impairment [11].

Two neural background tests were performed [12]. The Digit Span test (DS) was divided into forward memorization and reverse memorization. Each subject was tested for 3 min followed by 10 min of rest. The subjects repeated the numeric sequence read by the testers, reflecting short-term memory, working memory ability, and frontal lobe executive function. The Verbal Fluency Test (VFT) counted the number of animals that the subjects recited within a minute, reflecting the executive function of the frontal lobe [13]. Each subject completed the Verbal Fluency test in 1 min, followed by 5 min of rest.

**Figure 1:**
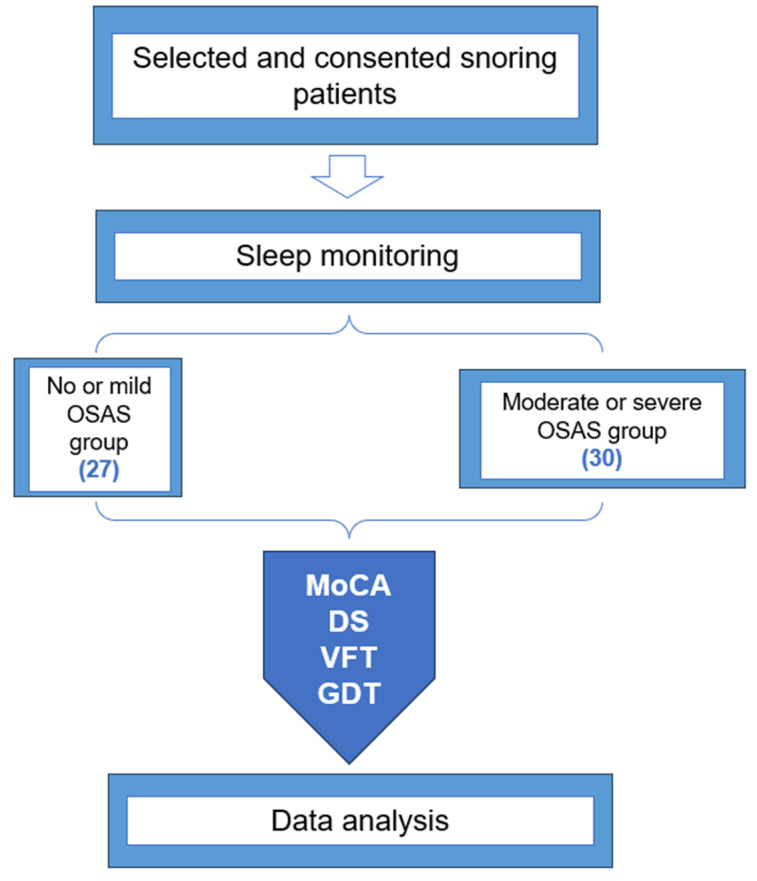
Flow chart of experimental design.

As for the decision-making function testing, the GDT is a 20 min cognitive test task that reflects the subjects’ decision-making ability when the risk probability is clear [14]. This test has traditionally been used to assess risky behavior in many different patient populations that range from generally healthy individuals to patients with neurological diseases such as Alzheimer’s Disease and mental illnesses such as binge eating disorder [15]. The computer simulates gambling by rolling dice. In this game, the subjects know the probability of risk. That is, the combination of probability and bet remains constant, and the subjects are provided CNY 1000 (roughly USD 137.90). The goal is to win as much money as possible by guessing the number that will appear on the computer screen. Participants are given a total of 18 guesses in which they can choose a single number, or a combination of two to four numbers (see Figure 1). As long as one of the selected combinations of numbers matches the number shown on the screen, the asset corresponding to the bet can be won [16]. The safety options showed smaller monetary bets and larger, more diverse numerical combinations; whereas the risk (or adverse) option showed higher monetary bets and fewer numerical combinations. The observation indicators are total assets, negative feedback utilization (negative feedback utilization = the number of times the risk option was converted to the safety option after losing money/the total number of times the risk option was selected), number of safety and risk options [16].

The results of GDT observation indicators are automatically generated by the software. The results of GDT, nocturnal sleep monitoring, demographic data, past medical history, medication and smoking history, answers to the questionnaires and questionnaire scores were properly collected by the research team.

### 2.4. Sleep Studies

Sleep monitoring was carried out in the sleep monitoring room of Department of Respiratory and Critical Medicine or Otorhinolaryngology Head and Neck Surgery. The subjects were connected to the monitor by standardized, qualified health care professionals, and were provided with a standardized explanation of the procedures with informed consent. Staff ensured the environment was comfortable and quiet to limit outside disturbances. Pulse oxygen saturation, apnea hypopnea index (AHI), times of obstruction, and hypopnea were recorded. Subjects were prohibited from taking sedative and hypnotic drugs and smoking, as well as consuming alcohol, tea, coffee, and other mind-altering substances for 24 h prior to the experiment. The patients’ usual habits in preparation for falling asleep determines the starting time of sleep monitoring. The total recording time is more than 7 h. The diagnostic criteria of OSAS are as follows: oral and nasal airflow ceases for more than 10 s throughout the entire monitoring process, 30 or more apneic events [reduction of airflow greater than or equal to 90% compared to the pre-event amplitude [8] or apnea-hypopnea index (AHI) ≥ 5 times/hour throughout testing. An AHI ≥ 15 times/hour is required for moderate and severe OSAS [9]. All patients completed cognitive tests on the night of sleep monitoring (Figure 1).

### 2.5. Statistical Analysis

SPSS 23.0 software was used for statistical analysis. The measurement data in accordance with the normal distribution are represented by *x ± s*, and when the corresponding conditions are met, the data statistics are carried out by two independent samples *t*-tests; the data of skewed distribution are represented by the median and quartile spacing, and the completely random designed rank sum test of two sample data is used for data statistics. The stepwise regression method of multiple linear regression was used for multivariate correlation analysis. The difference was statistically significant (*p* < 0.05).

## 3. Results

### 3.1. Participants

A total of 57 patients was selected into the study group. There were 30 patients with moderate and severe OSAS, including 27 males and 3 females, aged from 29 to 60 years old, with an average age of 45.50 ± 8.76 years. In the same period, 27 participants with no or mild OSAS were selected, including 19 males and 8 females, aged from 24 to 65 years old, with an average age of 44.48 ± 9.99 years. The patients in the group had no history of severe systemic diseases such as heart, lung, liver and kidney, no mental disorders and organic brain diseases affecting cognitive function, no substance abuse, and no central and mixed sleep apnea syndrome.

### 3.2. Comparison of General Data and Sleep Monitoring

A statistically significant difference (27.45, 31.13 vs. 23.06, 28.10, *p* = 0.002) was found between the body mass index (BMI) of patients with moderate to severe OSAS compared to that of patients with no or mild OSAS. The results of sleep breathing monitoring showed that the AHI value of moderate and severe OSAS group was higher than that of the no or mild OSAS group (25.15, 56.28 vs. 3.10, 10.50, *p* < 0.001), and the longest apnea time of moderate and severe OSAS group was longer than that of the no or mild OSAS group, and there was a significant difference between the two groups (92.13, 113.48 vs. 42.50, 95.90, *p* = 0.001). As for the change of nocturnal oxygen saturation, the change was more obvious in the moderate and severe OSAS group, where the average blood oxygen saturation (blood oxygen saturation, SaO2) was lower (92.33, 95.13 vs. 94.50, 96.40, *p* < 0.001) and the oxygen reduction index was higher (10.83, 33.55 vs. 0.60, 4.10, *p* < 0.001).

### 3.3. Montreal Cognitive (MoCA) Test Scale, Digit Span (DS) and Word Verbal Fluency Test (VFT)

When compared to the no or mild OSAS group, the moderate to severe OSAS group showed statistically lower values across each measured assessment: MoCA score (22, 24 vs. 23, 27, *p* = 0.001), the number of positive memorization of DS, the number of reverse memorization of DS (4, 5 vs. 5, 6, *p* = 0.004) and the number of VFT (12.27 ± 1.48 vs. 13.56 ± 2.49, *p* = 0.020). Among all the indexes of MoCA, the scores of visual spatial executive function test in patients with no or mild OSAS were higher than those in patients with moderate and severe OSAS (3, 4 vs. 2, 3, *p* < 0.001), but there was no significant difference in other cognitive areas (*p* > 0.05, Table 1 and Table 2).

### 3.4. Game of Dice Tasks

**Figure 2:**
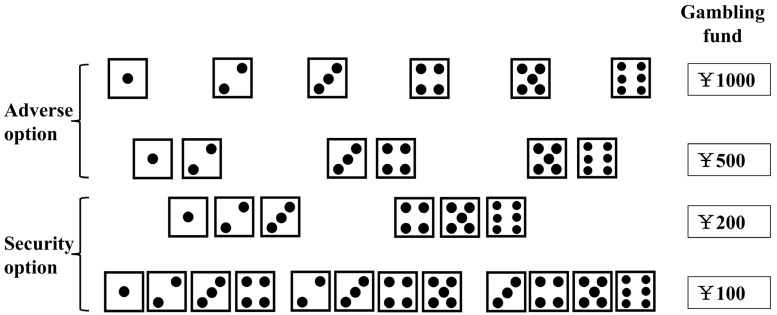
Schematic diagram of GDT instruction language.

**Figure 3:**
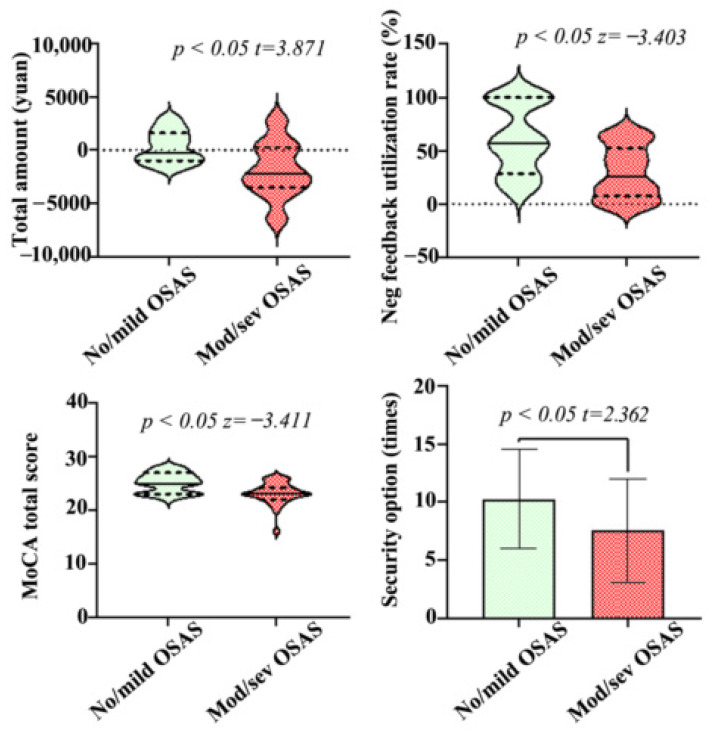
Comparison of total assets, negative feedback utilization, safety options and total score of MoCA between moderate and severe OSAS group and no or mild OSAS group in the GDT test.

In the Game of Dice Task (GDT), the total assets of the no or mild OSAS group was slightly higher than that of the moderate and severe OSAS group (300.00 ± 1509.97 vs. −1846.67 ± 2587.20, *p* < 0.001) (Figure 2 and Figure 3). The negative feedback utilization rate of the no or mild OSAS group was higher than that of the moderate and severe OSAS group (28.57, 100.00 vs. 7.50, 52.50, *p* = 0.001) (Figure 2 and Figure 3), and the difference between the two groups was statistically significant, that is, the patients in this group were more likely to choose the safety option next time after choosing the risk option to lose money. The moderate to severe OSAS group is more likely to choose the risk option than no or mild OSAS group (10.47 ± 4.43 vs. 7.74 ± 4.26, *p* = 0.022). The no or mild OSAS group was more likely to choose the safety option than moderate to severe OSAS group (10.26 ± 4.26 vs. 7.53 ± 4.43, *p* = 0.022) (Figure 2 and Figure 3, Table 3).

Figure 2—This figure is a visual representation of the GDT cognitive test task. It shows the adverse and security dice combinations as well as the corresponding gambling fund that can be won by the participants. The adverse options include the top two rows of dice in the figure which correspond to CNY 1000 and CNY 500 gambling funds. The bottom two rows of dice in the figure represents the security options and corresponds to CNY 200 and CNY 100 gambling funds. The safety (or security) options showed smaller monetary bets and larger, more diverse numerical combinations, whereas the risk (or adverse) option showed higher monetary bets and fewer numerical combinations.

Figure 3—This figure displays four graphs that are related to the GDT test. All four graphs compare the no or mild OSAS group to the moderate and severe OSAS group. The top left graph is the total assets in CNY and the top right graph is negative feedback utilization. The bottom left graph is moCA total score and the bottom right graph is the security (safety) option.

### 3.5. Multiple Linear Regression Analysis of Dice Game Experiment

The number of risk options in GDT were treated as the dependent variable, while negative feedback utilization, AHI, longest apnea time, average SaO2, MoCA score, and VFT score were treated as independent variables and analyzed by multiple linear regression. The results showed that the choice of risk option was negatively correlated with the utilization rate of negative feedback, and positively correlated with the number of words answered in the VFT, but not with AHI and the longest apnea time (*p* > 0.05, Table 4).

## 4. Discussion

According to Shu-Chin et al., executive function, alertness, attention, and memory ability of patients with OSAS are worse than those of normal subjects, which are mainly related to the damage of brain cells and hippocampus caused by sleep fragmentation, chronic hypoxia, and hypercapnia [4]. Our study found that moderate and severe OSAS patients have impaired function of decision-making with known risk probability, which is related to the low utilization of negative feedback during testing. In the basic cognitive test, the overall cognitive function of patients with moderate and severe OSAS was lower than that of patients with no or mild OSAS, especially in the aspect of visual spatial executive function. The Verbal Fluency Test (VFT) and Digit Span (DS) test reflecting the executive function of the frontal lobe were also worse in patients with moderate to severe OSAS than those with no or mild OSAS.

In the Game of Dice Task (GDT), we found that after the loss of money, the moderate and severe OSAS group is still more likely to choose the risk option than the no or mild OSAS group. That is, the utilization rate of negative feedback is lower, and the total assets at the end of the test are less or even tend to lean towards negative assets. A study on frontal cortex and perceptual function of decision-making shows that the frontal cortex is the cognitive processing center for the function of decision-making [17,18]. Our study analyzes the factors related to the selection of risk options and evaluates the reasons that affect the function of decision-making. The results of our study show that the selection of risk options is negatively related to the utilization rate of negative feedback, suggesting that moderate and severe OSAS patients do not choose safety options at the right time after losing money, resulting in low total assets.

There was a positive correlation between the frequency of choosing risk options and the results of VFT, results consistent with possible frontal lobe impairment of executive function in patients with OSAS. In the study on the changes of brain connectivity under the working memory challenge in patients with sleep apnea, it was found that the effect of OSAS on brain connectivity, or the basis of working memory performance, showed that the effective connectivity from the right frontal polar cortex (FPC) and the frontal middle to the left FPC in OSAS patients was lower than that in the control group [19]. Our study found that the MoCA score in patients with moderate to severe OSAS was significantly lower than that in patients with no or mild OSAS (see Figure 2). This indicates that patients with moderate to severe OSAS had cognitive impairment, a finding which was mainly reflected in the impairment of visual space executive function. The performance in orientation and attention was not significantly different from that in patients with no or mild OSAS. This result was consistent with the results of CROSS et al., whose study suggests that executive function may be the most impaired cognitive area in patients with OSAS [20]. Verbal Fluency Test and Digit Span tests were used to reflect the short-term memory, working memory ability, and frontal lobe executive function of patients with OSAS [21]. The results of the two tests showed that the patients with moderate and severe OSAS were lower than those with no or mild OSAS, indicating that frontal lobe executive functioning was impaired in the moderate to severe OSAS group. The cognitive process corresponding to executive function is mainly concentrated in the cerebral cortex.

It has been found that OSAS causes cerebral cortex thinning which is thought to represent neuronal degeneration and loss of glial cells. Intermittent hypoxia, blood perfusion damage, and inflammation are some of the major risk factors contributing to these anatomical changes. This cascade eventually leads to neurocognitive changes [22]. At present, most studies suggest that nocturnal hypoxia is associated with mild cognitive impairment in patients with OSAS [23]. We found that patients with moderate to severe OSAS had longer maximum apnea time and lower mean oxygen saturation than patients with no or mild OSAS. Intermittent hypoxia occurs in patients with OSAS at night. Injury and endothelial cell stimulation during hypoxia and reoxygenation can cause excessive production of reactive oxygen species, which can induce or aggravate oxidative stress [24]. Areas of the brain, such as the prefrontal lobe, frontal cortex, basal ganglia, and hippocampus, are particularly vulnerable to prolonged hypoxic-ischemic damage, resulting in corresponding cognitive impairment [25]. Evaluation of functional magnetic resonance imaging (fMRI) studies show that sleep apnea is associated with reduced activation and connectivity in most task-specific neural networks [19]. The vulnerability of the prefrontal cortex has been demonstrated in most verbal working memory tasks. To summarize, moderate and severe OSAS can lead to cognitive impairment, reduced function of decision making, and risk selection is negatively correlated to negative feedback utilization.

There may be prefrontal cortex damage in patients with moderate and severe OSAS [26]. However, there are some shortcomings in this study, such as the lack of data based on imaging. We specifically chose to utilize questionnaires (such as MoCA) in this study due to the decreased accessibility of fMRI, we acknowledge that imaging studies in future research would allow for a more accurate and precise conclusion of injured tissue. We also acknowledge that the Verbal Fluency Test has been widely debated as a valid assessment of executive function throughout the scientific community. As such, we understand that this test does exhibit a large verbal component and thus may not adequately reflect isolated executive dysfunction. In future studies, we would like to explore more concrete methods of assessing cognitive function without severely decreasing the accessibility of our study to potential participants. Unfortunately, our study has does suffer from a small sample size given the low awareness of OSAS in the general population. We are hoping this study can highlight the need for continued participation and awareness resulting in future studies with larger sample sizes and increased power.

In the future, functional magnetic resonance imaging (fMRI) should be performed in patients with OSAS to further identify the area of brain injury in patients, infer the causes of cognitive impairment in OSAS, and to explore the mechanism of cognitive changes [27]. Secondly, for patients with moderate and severe OSAS, the recommended treatment is continuous positive airway pressure (CPAP) [28]. Some studies have suggested that long-term effective CPAP therapy can improve patients’ psychomotor alertness, attention and other cognitive functions [29]. This study did not observe the changes of cognitive function of moderate and severe OSAS patients after intervention, nor were sleep related issues explored. In the future, we plan to study the cognitive scale and fMRI changes of moderate and severe OSAS patients after intervention in efforts to better guide the diagnosis and treatment of the disease, provide early intervention and improve the prognosis to improve the quality of life of patients with OSAS [30]. Furthermore, we recognize that our study reflects a small sample. Future research would involve a larger sample size to more accurately reflect the impact of OSAS on cognitive functioning.

In this study, by using multiple linear regression analysis with the number of risky options as the dependent variables, it has been found that the difference between negative feedback utilization and VFT was statistically significant. Interestingly, it has further been found that negative feedback utilization was negatively correlated with the number of risky options; conversely, VFT was positively correlated with the number of risky options. Compared with negative feedback utilization, the effect size of lexical fluency was several times higher. Therefore, we are confident that the index of VFT has higher guiding value than other indicators in predicting damage to frontal lobe executive functions. It is necessary to point it out that, due to the small sample size and limited covariates, our conclusion should be interpreted with caution in generalized population. We are actively performing anther study with adequate randomized controls and larger sample size as well as more covariates to expand the present study.

It will be necessary to point it out that it will be remarkably interesting to include features of metabolic syndrome such as hypertension, hyperglycemia and hyperlipidemia in our study. We are actively expanding our study to address this.

## 5. Conclusions

In summary, this study found that patients with moderate to severe OSAS had impaired risk decision making and impaired executive functioning closely related to frontal lobe function, while memory, attention, language, abstraction, and orientation were relatively preserved. This conclusion is based on the use of MoCA’s subscales as field indicators. Specific in-depth tests were not used to assess the cognitive domains.

## Figures and Tables

**Table 1:** Comparison of general data and sleep monitoring index between moderate and severe OSAS group and no or mild OSAS group. A *t*-test was used to compare normal distributed continuous variables and the rank sum test was used to compare continuous variables that are not normally distributed.

Group	No or Mild OSA Group	Moderate and Severe OSA Group	*t/z*	*p*
*n* (Number of people)	27	30		
Age (years)	44.48 ± 9.99	45.50 ± 8.76	−0.410	0.683
Education (years)	11 (8, 16)	8 (7, 14)	−1.036	0.300
BMI (kg/m^2^)	25.50 (23.06, 28.10)	29.30 (27.45, 31.13)	−3.101	0.002
AHI (times/hour)	5.80 (3.10, 10.50)	43.40 (25.15, 56.28)	−6.473	<0.001
Maximum apnea time (s)	78.30 (42.50, 95.90)	104.10 (92.13, 113.48)	−3.300	0.001
Lowest SaO_2_ (%)	83.00 (51.00, 89.00)	61.50 (51.75, 80.25)	−1.315	0.188
Average SaO_2_ (%)	95.30 (94.50, 96.40)	93.80 (92.33, 95.13)	−3.742	<0.001
Oxygen reduction index	2.10 (0.60, 4.10)	20.25 (10.83, 33.55)	−5.739	<0.001

**Table 2:** Comparison of background cognitive function between moderate and severe OSAS group and no or mild OSAS group. A *t*-test was used to compare normal distributed continuous variables and the rank sum test was used to compare continuous variables that are not normally distributed.

Group	No or Mild OSA Group	Moderate and Severe OSA Group	t/z	*p*
MoCA Total score (score)	25 (23, 27)	23 (22, 24)	−3.411	0.001
Visual space executive function (score)	4 (3, 4)	3 (2, 3)	−4.409	<0.001
Delayed recall (score)	2 (2, 4)	2 (2, 3)	−0.680	0.497
Attention (score)	6.00 (6.00, 6.00)	6.00 (5.75, 6.00)	−0.088	0.930
Vocabulary (score)	2 (2, 3)	2 (1, 3)	−1.778	0.075
Abstract (score)	1 (0, 2)	1 (0, 2)	−0.662	0.508
Directional force (score)	6 (6, 6)	6 (6, 6)	−0.921	0.357
DS Positive direction (Number)	8 (7, 8)	6 (6, 7)	−4.020	<0.001
DS Reverse (Number)	5 (5, 6)	5 (4, 5)	−2.910	0.004
VFT (Number)	13.56 ± 2.49	12.27 ± 1.48	2.405	0.020

**Table 3:** Results of GDT in moderate and severe OSAS group and no or mild OSAS group. A *t*-test was used to compare the normal distributed continuous variables, and the rank sum test was used to compare continuous variables that are not normally distributed.

Group	*n*	Total Amount (CNY)	Negative Feedback Utilization Rate (%)	Safety Option (Times)	Risk Option (Times)
No or mild OSAS group	27	300.00 ± 1 509.97	57.14 (28.57, 100.00)	10.26 ± 4.26	7.74 ± 4.26
Moderate and severe OSAS group	30	−1846.67 ± 2587.20	25.40 (7.50, 52.50)	7.53 ± 4.43	10.47 ± 4.43
*t/z*		3.871	−3.403	2.362	−2.362
*p*		<0.001	0.001	0.022	0.022

**Table 4:** Multi-factor regression Analysis of risk options. Multifactor linear regression is used to study the impact of different variables on outcomes.

Project	*β (95% Confidence Interval)*	Std. Error	*t*	*p*
Risk option (Constant)	6.050 (0.782, 11.317)	2.627	2.303	0.025
Negative feedback utilization rate	−0.098 (−0.123, −0.072)	0.013	−7.539	<0.001
MoCA Total score	−0.307 (−0.730, 0.117)	0.211	−1.455	0.152
VFT	0.581 (0.184, 0.978)	0.198	2.936	0.005
AHI	−0.018 (−0.074, 0.037)	0.028	−0.664	0.509
Maximum apnea time	0.022 (−0.010, 0.054)	0.016	1.397	0.168
Average SaO_2_	0.184 (−0.385, 0.753)	0.283	0.650	0.519

## Data Availability

The data generated during and/or analyzed during the current study are available from the corresponding authors on reasonable request.
